# Inflammation Triggers RNA Transfer from Blood Cells to Brain Neurons

**DOI:** 10.1371/journal.pbio.1001875

**Published:** 2014-06-03

**Authors:** Richard Robinson

**Affiliations:** Freelance Science Writer, Sherborn, Massachusetts, United States of America

The nervous and immune systems are the two most complex systems in the body. That complexity is amplified by the fact that each influences the other, as they constantly exchange messages in response to environmental and internal cues. The best known messages from immune cells are the cytokines, which trigger changes within neurons through well-established receptor-initiated cascades. But in a new study in this issue of *PLOS Biology*, Kirsten Ridder, Stefan Momma, and colleagues show that hematopoietic cells, which include the cells of the immune system, release messenger RNAs (mRNAs) and microRNAs (miRNAs) that are absorbed by neurons. This mechanism of signaling is elevated in response to inflammation, and the transferred RNAs have the potential to influence neuronal responses.

Inflammation has recently been shown to drive an even more unexpected neuro–immune interaction—fusion of hematopoietic cells with Purkinje neurons of the cerebellum, in which the resulting cell contains both nuclei. The authors sought to determine whether less drastic transfers of genetic messages might be taking place as well.

To do so, they relied on the Cre recombination system. In this system, a gene of interest is engineered to be flanked by target sequences (“lox” sequences) for the enzyme Cre recombinase. Enzymatic action rearranges the site and triggers expression of the gene within. Here, the authors used mice with a lox-flanked reporter gene in all tissues. The Cre recombinase gene, though, was introduced only to hematopoietic cells. Thus, any non-hematopoietic cells expressing the reporter gene must have picked up either the Cre recombinase gene, its mRNA, or the protein itself, from hematopoietic cells.

The authors found that a small number of Purkinje neurons expressed the reporter, despite the absence of any double nuclei (ruling out cell fusion as described above and therefore gene transfer). So how were the neurons getting the message? Extracellular vesicles (EVs) have lately emerged as a significant vehicle for trafficking multiple kinds of molecules between cells, including proteins and mRNAs, so the authors isolated EVs from peripheral blood. When they analyzed the contents, they found the EVs contained Cre recombinase mRNA, but not the protein.

In healthy mice, the number of Cre-recombined neurons was very low, far less than 1%. But when the authors triggered a peripheral inflammatory response that number jumped by several orders of magnitude, affecting an average of 6% of Purkinje neurons. Under such conditions of inflammation, several other types of brain neurons were also targeted, including those in the hippocampus, cortex, and substantia nigra.

These results show that mRNA can be transferred from immune cells of the peripheral blood to neurons of the central nervous system via EVs. It remains to be seen whether other immune-derived mRNAs take advantage of the same means of transport to enter neurons.

But known exosome passengers also include miRNAs, short non-coding RNAs that influence gene expression in many types of organisms. By excising single neurons, the authors showed that Cre-recombined neurons contained three miRNAs not found in non-recombined neurons; the same three were also present in exosomes isolated from blood in mice experiencing inflammation. These results suggest that the three miRNAs could derive from the same immune cells as the Cre recombinase. If that is so, this study indicates there is not only another medium of information exchange between the immune and nervous system but also a whole new type of message, and it suggests that the regulation of the nervous system by the immune system may be more extensive and intimate than previously appreciated.

**Figure:**
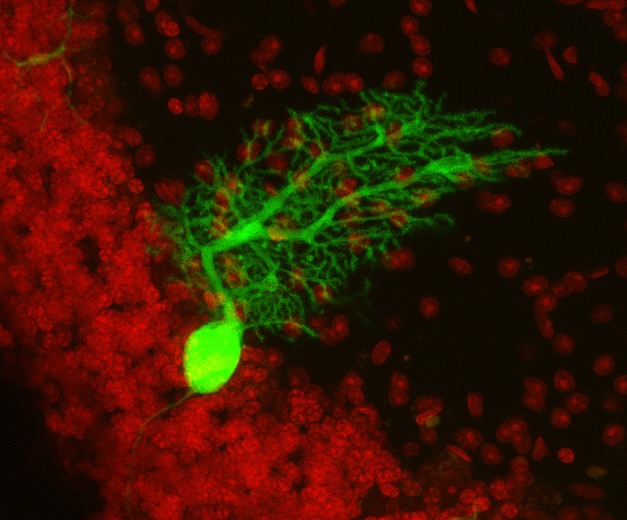
Purkinje neurons express a reporter gene upon uptake of RNA from hematopoietic extracellular vesicles. The number of these vesicle-targeted cells increases in response to inflammation. *Image Credit: Stefan Momma, Jadranka Macas*

**Ridder K, Keller S, Dams M, Rupp A-K, Schlaudraff J, et al. (2014) Extracellular Vesicle-Mediated Transfer of Genetic Information Between the Hematopoietic System and the Brain in Response to Inflammation.** doi:10.1371/journal.pbio.1001874.

